# Construction and characterization of new *piggyBac *vectors for constitutive or inducible expression of heterologous gene pairs and the identification of a previously unrecognized activator sequence in *piggyBac*

**DOI:** 10.1186/1472-6750-7-5

**Published:** 2007-01-18

**Authors:** Xianzong Shi, Robert L Harrison, Jason R Hollister, Ahmed Mohammed, Malcolm J Fraser, Donald L Jarvis

**Affiliations:** 1 Department of Molecular Biology University of Wyoming 1000 E. University Avenue Laramie, WY, USA 82071; 2 Chesapeake-PERL, Inc. 8510A Corridor Rd Savage, MD, USA 20763; 3 Department of Biological Sciences University of Notre Dame Notre Dame, IN, USA 46556-0369; 4 USDA, ARS, PSI Insect Biocontrol Lab Building 011A, Room 214, BARC-W 10300 Baltimore Ave.Beltsville, MD, USA 20705; 5 USDA, ARS, NAA, PIADC Plum Island Animal Disease Center P.O. BOX 848, GREENPORT, LI Orient Point, NY, USA 11944

## Abstract

**Background:**

We constructed and characterized several new *piggyBac *vectors to provide transposition of constitutively- or inducibly-expressible heterologous gene pairs. The dual constitutive control element consists of back-to-back copies of a baculovirus immediate early (*ie1*) promoter separated by a baculovirus enhancer (*hr5*). The dual inducible control element consists of back-to-back copies of a minimal cytomegalovirus (*CMV*_*min*_) promoter separated by a synthetic operator (*TetO7*), which drives transcription in the presence of a mutant transcriptional repressor plus tetracycline.

**Results:**

Characterization of these vectors revealed an unexpected position effect, in which heterologous genes adjacent to the 3'- terminal region ("rightward" genes) were consistently expressed at higher levels than those adjacent to the 5'-terminal region ("leftward" genes) of the *piggyBac *element. This position effect was observed with all six heterologous genes examined and with both transcriptional control elements. Further analysis demonstrated that this position effect resulted from stimulation of rightward gene expression by the internal domain sequence of the 3'-terminal region of *piggyBac*. Inserting a copy of this sequence into the 5'- terminal repeat region of our new *piggyBac *vectors in either orientation stimulated leftward gene expression. Representative *piggyBac *vectors designed for constitutive or inducible expression of heterologous gene pairs were shown to be functional as insect transformation vectors.

**Conclusion:**

This study is significant because (a) it demonstrates the utility of a strategy for the construction of *piggyBac *vectors that can provide constitutive or inducible heterologous gene pair expression and (b) it reveals the presence of a previously unrecognized transcriptional activator in *piggyBac*, which is an important and increasingly utilized transposable element.

## Background

*piggyBac *is a class II transposable element that was originally discovered as the IFP2 element from the lepidopteran insect cell line, TN-368, due to its tendency to insertionally inactivate certain baculovirus genes to produce mutants with a distinctive plaque phenotype known as Few Polyhedra [1,2]. Functionally, *piggyBac *encodes a transposase with a precise cut and paste mechanism and a unique preference for TTAA sites [3-6]. Thus, it is considered to be the type element of the TTAA-specific transposon family [7]. Structurally, *piggyBac *is a 2.4 kb DNA molecule with a single 1.8 kb open reading frame that terminates on both ends with 13 bp perfect inverted terminal repeat domains (TRD's). *piggyBac *also has two additional 19 bp subterminal inverted repeats located asymmetrically 31 bp from the 5'-TRD and 3 bp from the 3'-TRD [4]. Sequences similar to the *piggyBac *open reading frame have been identified in all animal species for which extensive genomic sequences are available, including the human. However, most appear to be either incomplete or interrupted and, therefore, probably do not encode functional transposons [8].

The mobility and transposition functions of *piggyBac *have been established and exploited to develop an important binary system for insect germline transformation [3]. This system consists of a DNA vector, which can be mobilized due to the presence of the *piggyBac *5'- and 3'-TRD sequences, and a helper plasmid, which encodes the transposase. The vector also includes a promoter, which controls transcription of an inserted, heterologous gene of interest, and a whole-body or eye color marker, which can be used to identify transgenic offspring.

The Mediterranean fruit fly was the first target organism to be successfully transformed using the *piggyBac *system [9] and it has subsequently been used to transform a wide variety of insects (reviewed in reference [10]). Recently, the *piggyBac *system has been used to transform many other types of organisms ranging from the protist, *Plasmodium falciparum *[11] to the mouse, *Mus musculus *[12]. Thus, *piggyBac *is widely and increasingly recognized as an important tool for genetic transformation in many different biological systems.

Current *piggyBac *vectors are designed to introduce a single heterologous gene of interest into the genome of a target organism, in addition to the marker gene. However, some transgenic approaches require the introduction of multiple heterologous genes of interest into a single target organism. To meet this requirement, we constructed a new set of *piggyBac *vectors designed to simultaneously transfer pairs of heterologous genes placed under the control of dual constitutive or regulated transcriptional elements, which included duplicate promoters in a back-to-back configuration. One advantage of this approach is that it allowed us to couple both promoters to a single enhancer or regulatory domain, which minimized the overall size of the transcriptional control region.

During the process of characterizing the induction of heterologous gene expression in lepidopteran insect cells by these new *piggyBac *vectors, we discovered an unexpected position effect, in which the rightward-oriented heterologous genes were consistently expressed at higher levels than the leftward-oriented ones. Further analysis revealed that this was the result of stimulation by a previously unrecognized activator element in the 3'-TRD of *piggyBac*. We subsequently duplicated this activating sequence and used it to balance expression of the rightward and leftward heterologous genes in our new vectors. Thus, this study yielded not only a substantial set of new *piggyBac *vectors, but also provided new basic information on this important transposable element, both of which will be of general interest to the biomedical research community.

## Results and Discussion

### New piggyBac vectors for transfer and constitutive or inducible expression of heterologous gene pairs

**Figure 1:**
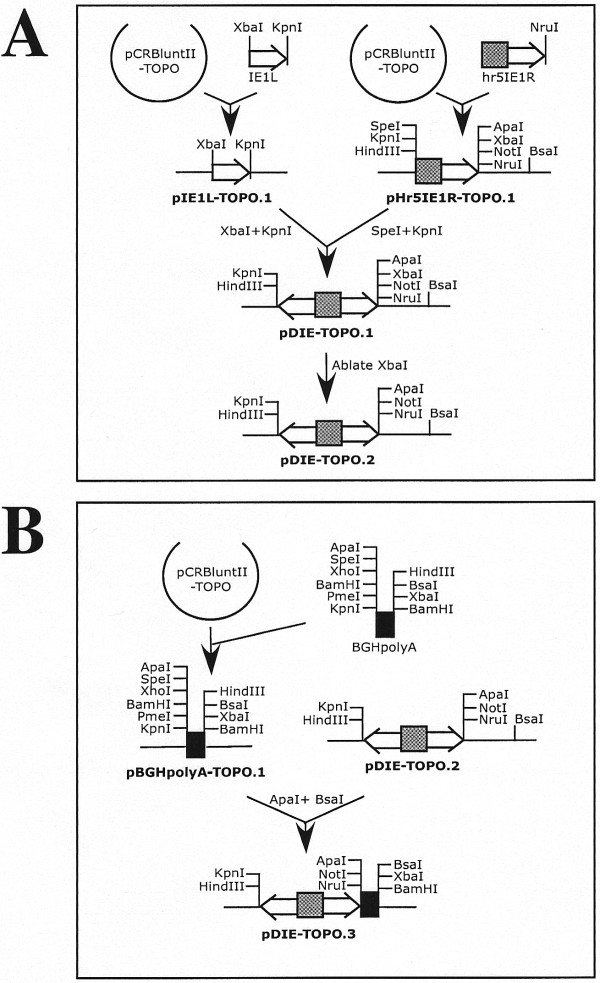
***piggyBac *vector constructions**. A. Construction of the dual constitutive transcriptional control element. B. Addition of a polyadenylation signal.

**Figure 2:**
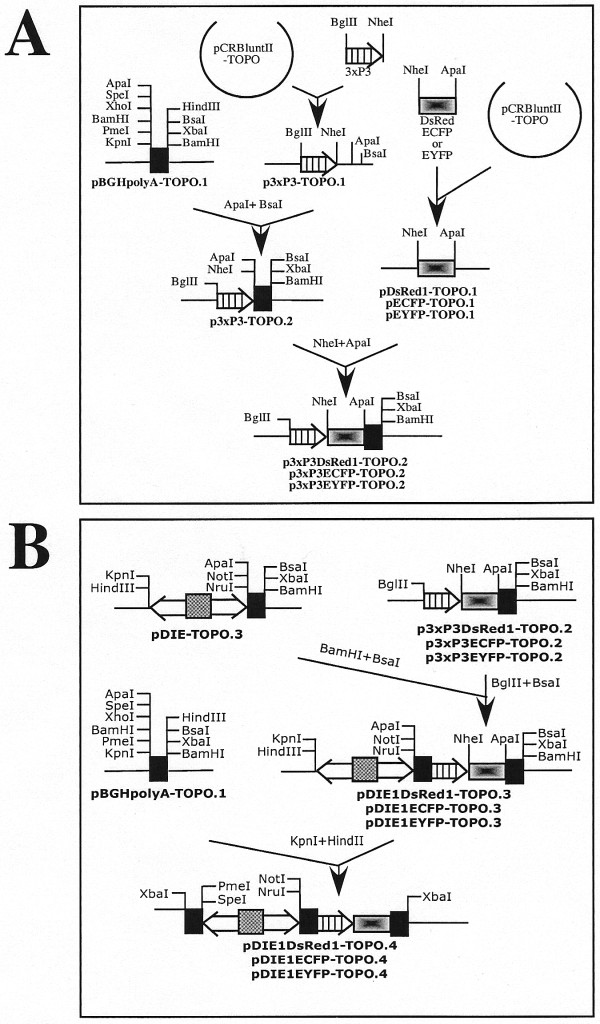
***piggyBac *vector constructions (continued)**. A. PCR amplification of the fluorescent eye color markers. B. Assembly of intermediate plasmids that include the dual constitutive transcriptional control element, fluorescent eye color markers, and appropriate polyadenylation sites.

**Figure 3:**
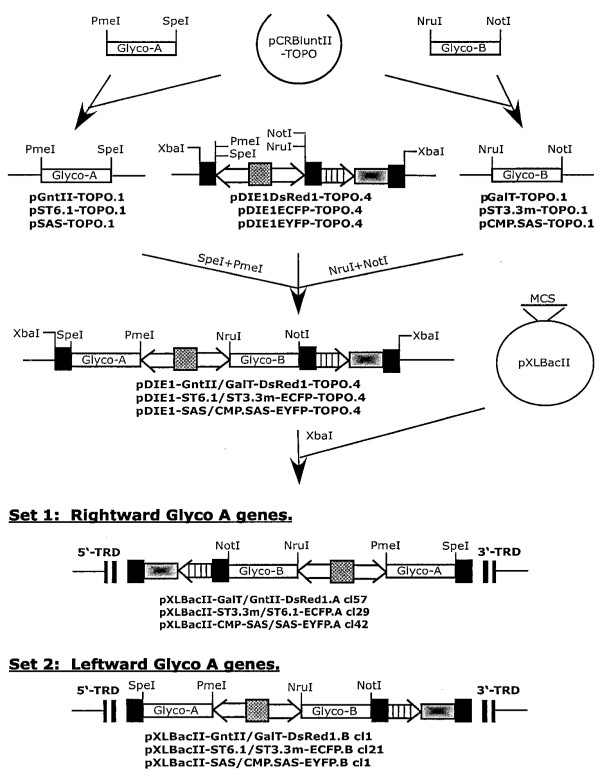
***piggyBac *vector constructions (continued)**. PCR amplification of six genes encoding mammalian glycosylation enzymes and their subsequent insertion into the key intermediate plasmids to produce the constitutive (*ie1-hr5-ie1*) dual *piggyBac *vectors.

**Figure 4:**
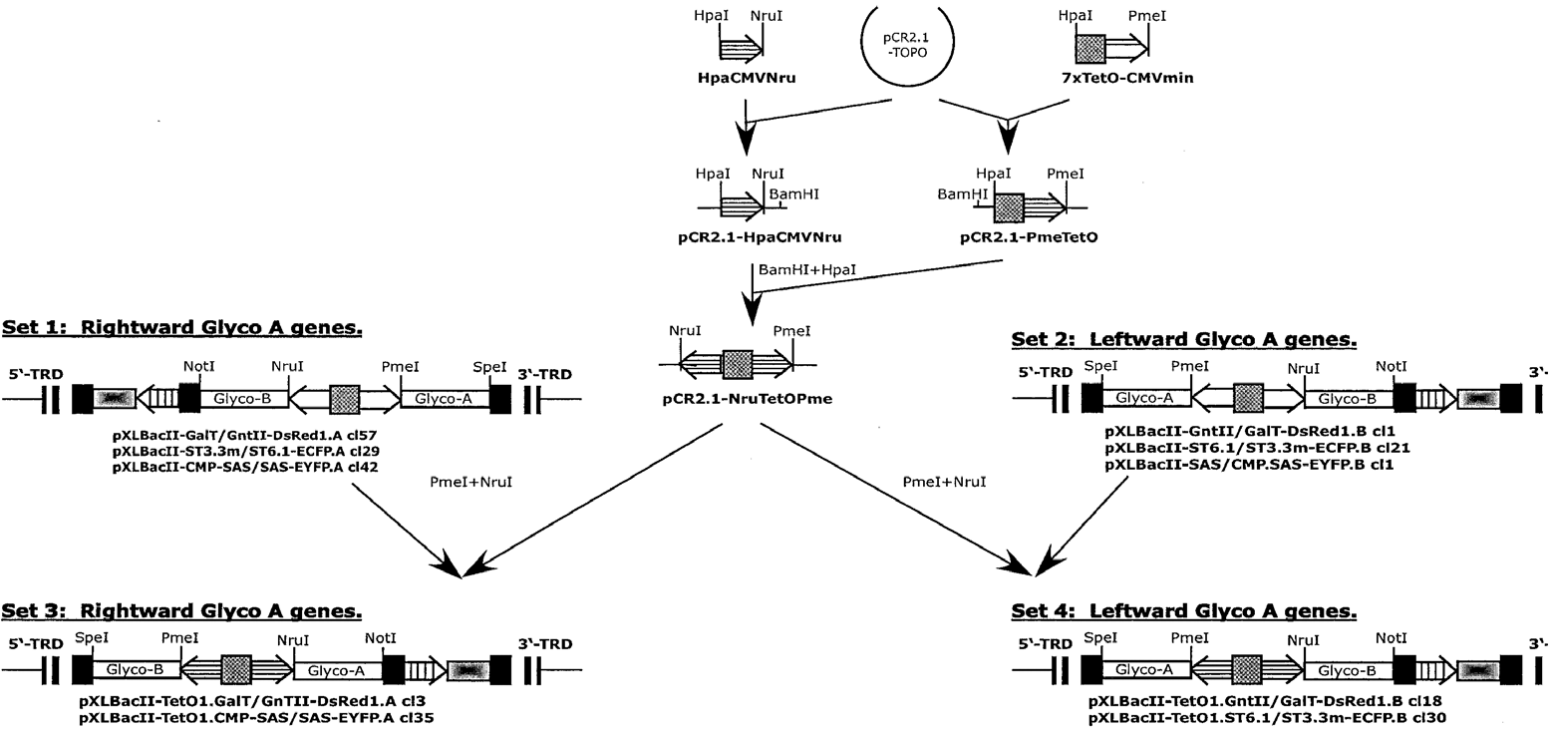
***piggyBac *vector constructions (continued)**. Replacement of the constitutive transcriptional control element to produce the inducible (*P*_*CMVmin*_-TetO7-*P*_*CMVmin*_) dual *piggyBac *vectors.

Previous studies have established that the addition of six mammalian genes can effectively humanize the protein *N*-glycosylation pathway of Sf9, a lepidopteran insect cell line (reviewed in references [13,14]). One of our current projects is designed to extend those studies by using these same genes to humanize the protein *N*-glycosylation pathway in an intact, multicellular lepidopteran insect. The *piggyBac *vector system was an obvious tool to use for this purpose. However, we were concerned about the efficacy of an attempt to use six separate vectors and, realizing that there were no *piggyBac *vectors that could be used to simultaneously transform an insect with multiple genes, we decided to construct a new set of vectors that could be used to transform a target organism with pairs of heterologous genes. We had previously designed and constructed plasmid vectors containing a transcriptional control element consisting of two back-to-back baculovirus immediate early gene (*ie1*) promoters separated by a baculovirus (*hr5*) enhancer [15,16]. In addition, we had inserted pairs of heterologous genes into these vectors and used the resulting constructs to transform lepidopteran insect cell lines independently of *piggyBac *and to isolate derivatives that constitutively expressed both heterologous genes [15,16]. Thus, we chose to construct a new set of *piggyBac *vectors in which pairs of heterologous genes could be placed under the control of the dual *ie1-hr5-ie1 *control element. We also considered that producing viable or fertile transgenic insect lines that constitutively express mammalian *N*-glycan processing genes might not be possible. Therefore, we constructed a second new set of *piggyBac *vectors in which pairs of heterologous genes could be placed under the control of a mosaic, tetracycline-inducible control element, which consisted of two back-to-back copies of the minimal human cytomegalovirus immediate early gene promoter (*P*_*CMVmin*_) separated by an operator (*TetO7*). This transcriptional control element had been shown to provide tetracycline-inducible gene expression in an insect system [17]. In addition to these transcriptional elements and heterologous gene pairs, each of these new *piggyBac *vectors also contained one of three different fluorescent protein-encoding genes under the control of an insect eye-specific promoter (*3xP3*; Horn, 2000 #1379], which could be used to identify transgenic offspring. The details of the cloning schemes used to construct the new *piggyBac *vectors described in this study are given in Materials and Methods and the cloning schemes and key genetic features of the new vectors are shown diagrammatically in Figs. 1, 2, 3, 4. The structure of each vector was analyzed in detail by restriction mapping, PCR, and/or DNA sequencing, as described in Materials and Methods. Subsequently, transient expression assays were performed to examine their functionality.

### An unexpected position effect on heterologous gene expression by the new piggyBac vectors

The molecular cloning schemes used in this study initially yielded four sets of *piggyBac *vectors in which six different heterologous genes were placed under the control of the constitutive or inducible regulatory elements described above (Fig. 4). Six of these vectors had the three "Glyco-A" genes (GnTII, ST6GalI, and SAS) with a rightward orientation (Fig. 4, Sets 1 and 3), while the other six had these same genes with a leftward oriention (Fig. 4, Sets 2 and 4) with respect to the 5'- and 3'-TRD elements of *piggyBac*, as defined by Li and coworkers [18].

**Figure 5:**
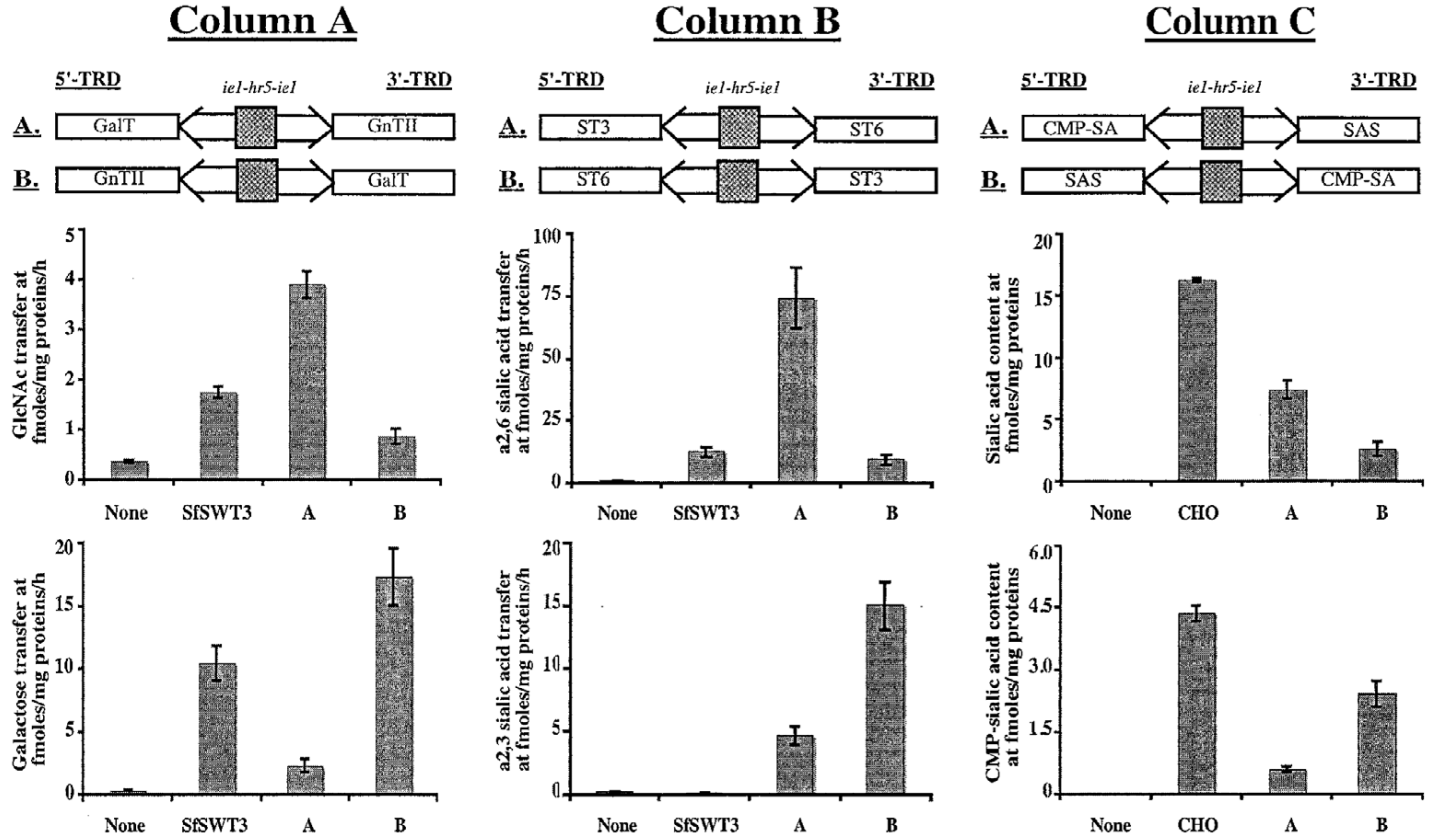
**Expression levels induced by constitutive *piggyBac *vectors**. The genetic structures of the different constitutive *piggyBac *vectors assayed in this experiment, with the differences in orientation of the heterologous genes indicated by A and B, are shown above the plots in Columns A, B, and C. Column A shows the GlcNAcTII (upper plot) and GalT (lower plot) activities induced by the vectors encoding these enzymes in orientations A and B. Column B shows the ST6GalI (upper plot) and ST3GalIII (lower plot) activities induced by the vectors encoding these enzymes in orientations A and B. Column C shows the sialic acid (upper plot) and CMP-sialic acid (lower plot) levels induced by the vectors encoding these enzymes in orientations A and B. The background levels in each assay were determined using extracts of mock-transfected Sf9 cells and are shown by the bars labeled "None". SfSWT3 and CHO refer to extracts of a transgenic insect cell line [15, 16] or Chinese hamster ovary cells, which served as positive controls for these assays.

**Figure 6:**
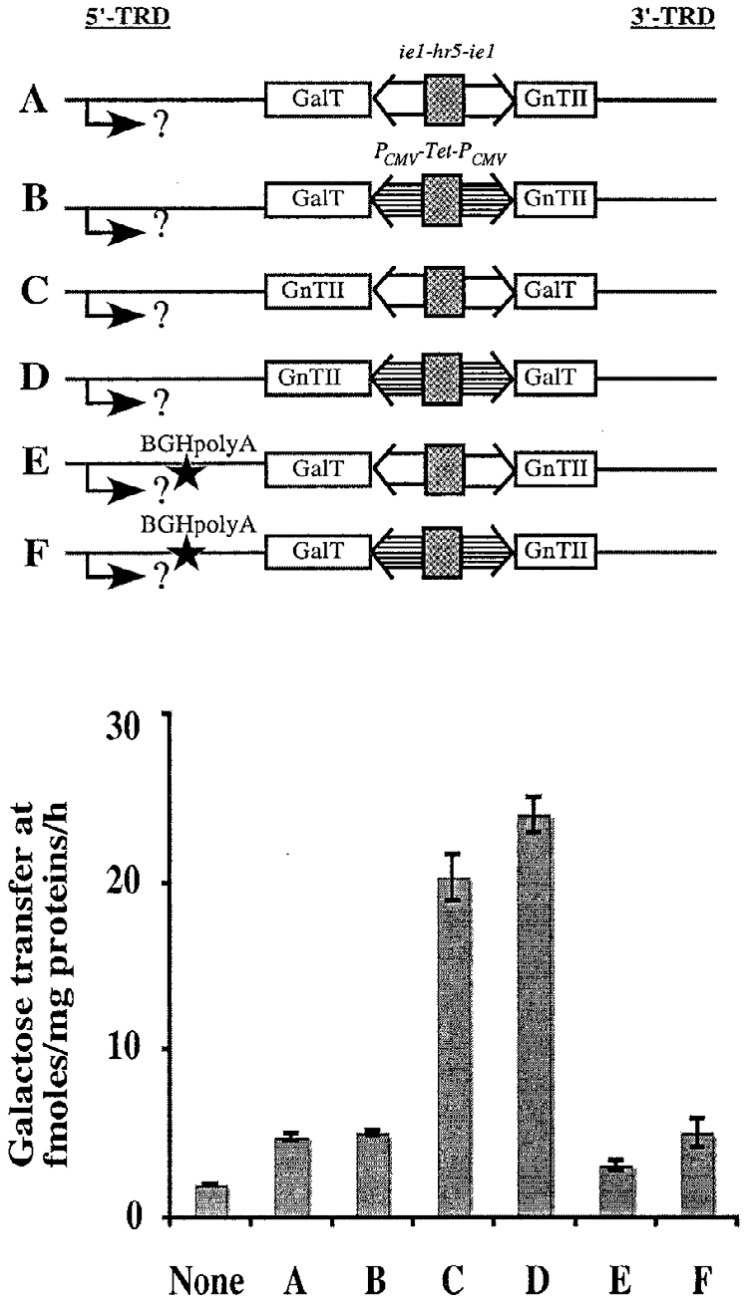
**Effects of introducing BGHPolyA to arrest potential antisense transcription**. The genetic structures of the different *piggyBac *vectors assayed in this experiment (A-F) are shown at the top, with the dual constitutive (*ie1-hr5-ie1*) transcriptional control elements indicated by open boxes, the dual inducible (*P*_*CMVmin*_-TetO7-*P*_*CMVmin*_) transcriptional control element indicated by horizontally striped boxes, the orientations of the various heterologous genes shown, and the newly introduced BGHPolyA site marked with a star. The rightward-oriented arrow marked with a question mark depicts the hypothesis that antisense transcription originating in the 5'-TRD region could down-regulate expression of the leftward-facing heterologous genes. The plot shows the relative GalT activity levels induced by each of the indicated *piggyBac *vectors, together with the background levels determined using extracts of mock-transfected Sf9 cells (None).

The first set of transient expression assays focused on the ability of the *piggyBac *vectors containing the dual constitutive transcriptional element (*ie1-hr5-ie1*) to induce heterologous gene expression. Unexpectedly, the results showed that the vectors containing the rightward heterologous genes induced higher levels than those containing the leftward genes (Fig. 5). For example, cells transfected with the *piggyBac *vector containing the GnTII gene in the rightward orientation (vector A in Column A, Fig. 5) had about 4.5X more GnTII activity than cells transfected with the *piggyBac *vector containing this same gene in the leftward orientation (vector B in Column A, Fig. 5). Similarly, vectors encoding GalT, ST6GalI, and ST3GalIII genes in the rightward orientation induced about 8X, 8X, and 3X more activity, respectively, than vectors encoding these genes in the leftward orientation (Fig. 5, columns A-B). This effect was not restricted to mammalian glycosyltransferase genes, as *piggyBac *vectors containing SAS and CMP-SAS genes in the rightward orientation induced about 3X and 4X higher sialic acid and CMP-sialic acid contents, respectively, than those containing these same genes in the leftward orientation (Fig. 5, Column C). Furthermore, this effect was not restricted to the dual constitutive control element (*ie1-hr5-ie1*), as it also was observed with *piggyBac *vectors containing heterologous gene pairs under the control of the inducible control element (*P*_*CMVmin*_-*TetO7*-*P*_*CMVmin*_; compare bars B and D in Fig. 6). These results revealed that the new *piggyBac *vectors constructed in this study exhibited a strong position effect, in which heterologous genes cloned in the rightward orientation were uniformly induced at higher levels than the same genes cloned in the leftward orientation, irrespective of the identity of the heterologous gene or control element used for its expression.

### The position effect does not reflect antisense down-regulation of the leftward genes

One hypothetical explanation for this striking position effect was that the *piggyBac *transposase promoter located in the 5'-TRD of the *piggyBac *vectors, which is downstream and in opposite orientation of the leftward genes, produced antisense transcripts that down-regulated expression of these leftward genes. Experiments were designed and performed to address this possibility. If the position effect observed with the new *piggyBac *vectors reflected down-regulation of the leftward genes by antisense transcription originating in the downstream 5'-TRD, the introduction of a polyadenylation signal between the two transcription units should reduce or eliminate this effect. The new polyadenylation signal would be expected to direct cleavage and polyadenylation of transcripts originating in the 5'-TRD, resulting in transcripts that would not overlap with the downstream region encoding the leftward heterologous gene in the opposite orientation. Thus, the newly inserted polyadenylation signal would be expected to block any potential negative effect arising from an antisense RNA mechanism. The two *piggyBac *vectors encoding constitutive or inducible GalT genes in the leftward orientation (Fig. 6E and 6F) were used as targets for the insertion of a BGHpolyA signal in the appropriate orientation downstream of the 5'-TRD. Subsequently, transient expression assays were performed to compare the GalT expression levels induced by these new *piggyBac *derivatives with those induced by the original vectors encoding the constitutive or inducible GalT genes in either the rightward or leftward orientations. The results confirmed that the original vectors encoding the rightward (Fig. 6, vectors C and D) GalT genes induced higher levels of GalT activity than those encoding the leftward (Fig. 6, vectors A and B) GalT genes. The results also showed that insertion of the BGHpolyA signal failed to reduce or eliminate this position effect, irrespective of promoter type (Fig. 6, vectors E and F).

**Figure 7:**
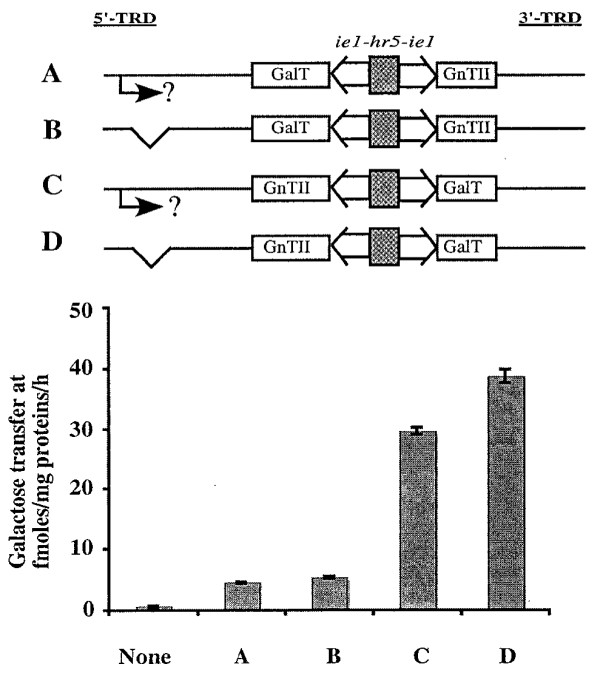
**Effects of deleting the transposase transcriptional control element in the 5'-TRD region**. The genetic structures of the constitutive *piggyBac *vectors assayed in this experiment (A-D) are shown at the top, with the orientations of the various heterologous genes shown, and the transposase promoter deletion indicated. The rightward-oriented arrow marked with a question mark depicts the hypothesis that antisense transcription originating in the 5'-TRD region could down-regulate expression of the leftward-facing heterologous genes. The plot shows the relative GalT activity levels induced by each of the indicated *piggyBac *vectors, together with the background levels determined using extracts of mock-transfected Sf9 cells (None).

Another way to reduce or eliminate a potential negative effect of antisense transcription on leftward gene expression was to delete the 5'-TRD internal domain sequence, which contains the transposase transcriptional initiation region [4]. Thus, this region was deleted from *piggyBac *vectors containing the constitutive GalT gene in the leftward or rightward orientations (Fig. 7, vectors B and D) and GalT expression levels induced by these new derivatives were compared to those induced by controls containing the intact promoter. Interestingly, the *piggyBac *vectors with the transposase promoter deletion induced about 20-30% higher GalT activity than the controls, irrespective of the orientation of the GalT gene (Fig. 7). However, the promoter deletion failed to reduce or eliminate the observed position effect, as the levels of GalT expression observed with the leftward gene remained lower than those observed with the rightward gene, even when the 5'-TRD internal domain was deleted.

Together, the results of the transient expression assays performed using the *piggyBac *vectors with BGHpolyA insertions or transposase promoter deletions strongly suggested that antisense transcription originating in the *piggyBac *transposase promoter within the 5'-TRD is not responsible for the observed position effect.

### The position effect reflects activation of the rightward genes

**Figure 8:**
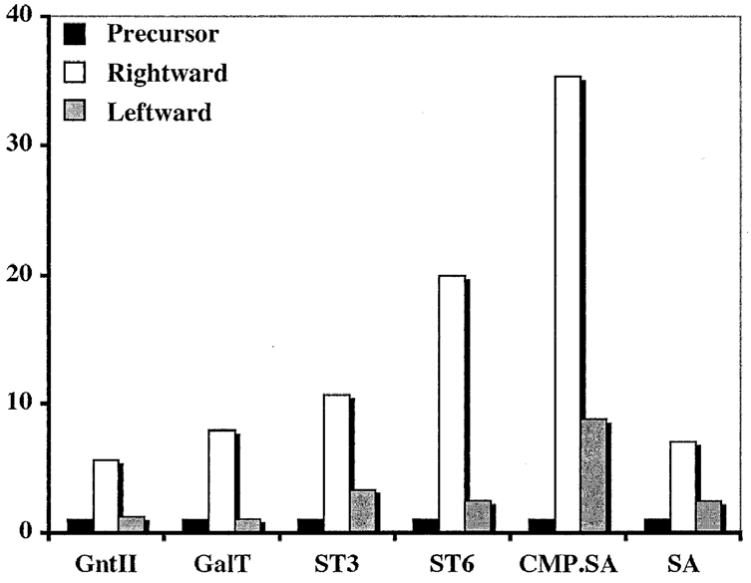
**Expression levels induced by precursor plasmids and constitutive *piggyBac *vectors**. The plot shows the levels of enzyme activity induced by precursor plasmids or *piggyBac *vectors encoding the six heterologous genes of interest under the control of the constitutive transcriptional control element. Black bars show the levels obtained with the precursor plasmids, white bars show the levels obtained with the *piggyBac *vectors containing heterologous genes in the rightward orientation, and gray bars show the levels obtained with the *piggyBac *vectors containing heterologous genes in the leftward orientation.

Another hypothetical explanation for the position effect observed with the new *piggyBac *vectors was that gene expression in the rightward direction is somehow activated relative to gene expression in the leftward direction. A preliminary clue indicating that this might be the correct hypothesis was obtained by comparing the levels of expression induced by *piggyBac *vectors containing the rightward or leftward heterologous genes with those induced by precursor (pCRBluntII-TOPO) plasmids containing the same genes outside the context of *piggyBac *(Fig. 8). The results of these assays revealed that the levels of activity induced by the precursor plasmids were more similar to those induced by the *piggyBac *vectors containing the leftward than the rightward heterologous genes. The activity levels induced by the *piggyBac *vectors containing the heterologous genes in the rightward orientation were significantly (5X to over 35X) higher. Thus, together with our previous results, these results suggested that the position effect observed with our new *piggyBac *vectors results from the activated expression of heterologous genes cloned in the rightward orientation, irrespective of the transcriptional control element. Additional experiments were designed to further examine this possibility.

### A previously unrecognized activator sequence in the piggyBac 3'-TRD

**Figure 9:**
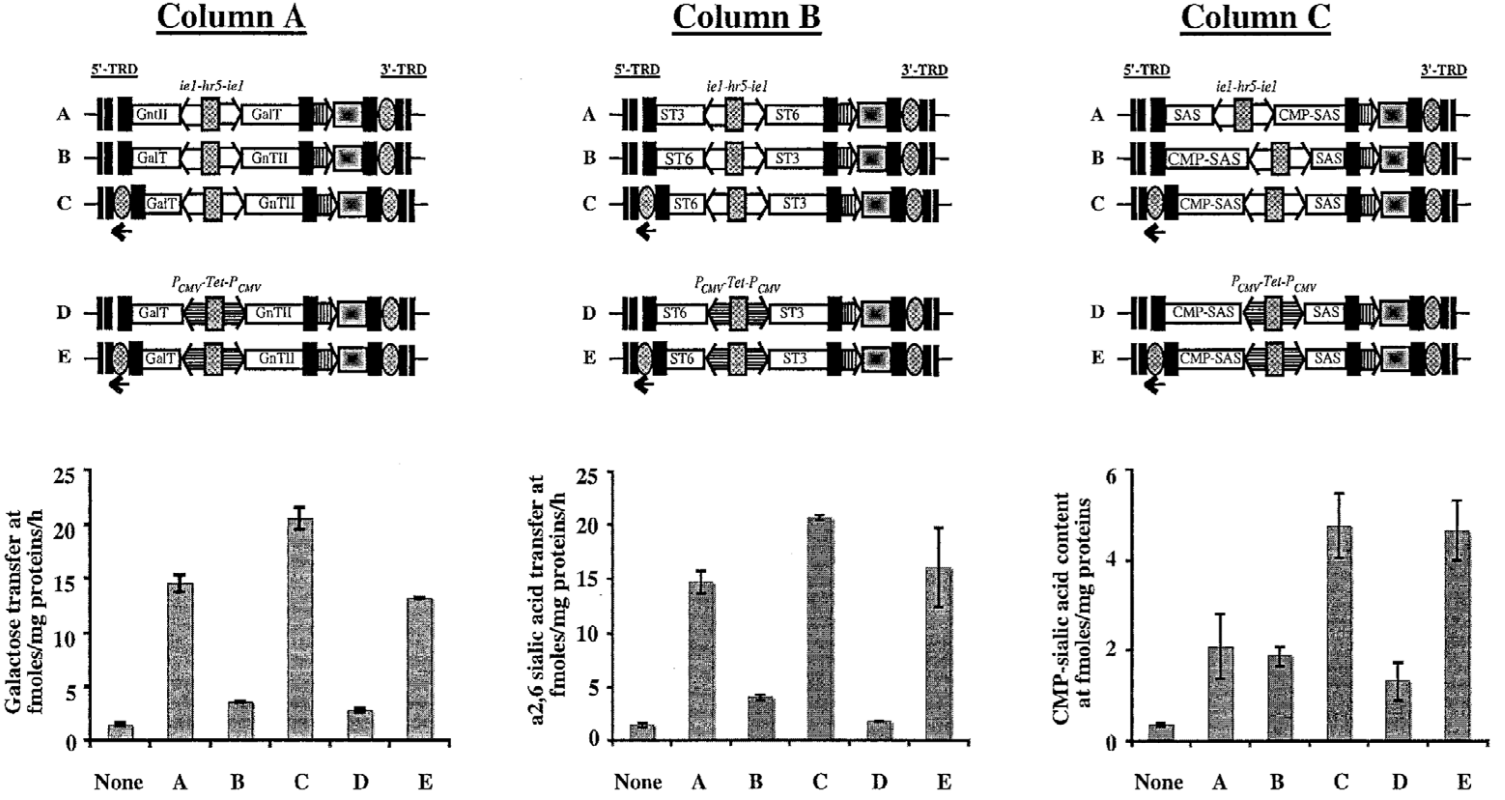
**Effects of introducing a copy of the 3'-TRD internal domain on leftward gene expression**. The genetic structures of the different *piggyBac *vectors assayed in this experiment (A-E) are shown above the plots in Columns A, B, and C. The dual constitutive (*ie1-hr5-ie1*) transcriptional control element is indicated by open boxes, the dual inducible (*P*_*CMVmin*_-TetO7-*P*_*CMVmin*_) transcriptional control element is indicated by horizontally striped boxes, the orientations of the various heterologous genes are shown, and the new 3'-TRD internal domain sequence introduced in the 5'-TRD region is marked with an cross-hatched oval and an arrow to show its orientation. The plot in Column A shows the GalT activities induced by the various GalT-encoding vectors A-E. The plot in Column B shows the ST6GalI activities induced by the various ST6GalI-encoding vectors A-E. The plot in Column C shows the amounts of CMP-sialic acid produced by the various CMP-SAS-encoding vectors A-E. The background levels in each assay were determined using extracts of mock-transfected Sf9 cells and are shown by the bars labeled "None".

The most obvious source of a sequence that might be able to activate expression of the rightward genes in our new *piggyBac *vectors was the downstream 3'-TRD, which includes the *piggyBac *3' terminal repeat and a 172 bp internal domain sequence with an 83% AT content [4,18]. The 3'-TRD internal domain sequence (3'-TRD_ID_) was targeted for further analysis. A PCR-amplified copy of this sequence was inserted downstream and in the same orientation with respect to the leftward facing constitutive and inducible genes into a selected subset of the *piggyBac *vectors produced in this study (Fig. 9, Columns A, B and C, constructs C and E). The enzyme activities induced by these new *piggyBac *derivatives, which contained the leftward-facing heterologous genes plus the 3'-TRD_ID _insert, were then compared to those induced by the original *piggyBac *vectors containing the same heterologous genes in either orientation, but without the additional 3'-TRD_ID _insert. The results of these assays showed that insertion of the putative activator sequence downstream of the leftward-oriented genes eliminated the position effect, irrespective of the identity of the heterologous gene or promoter type (Fig. 9).

**Figure 10:**
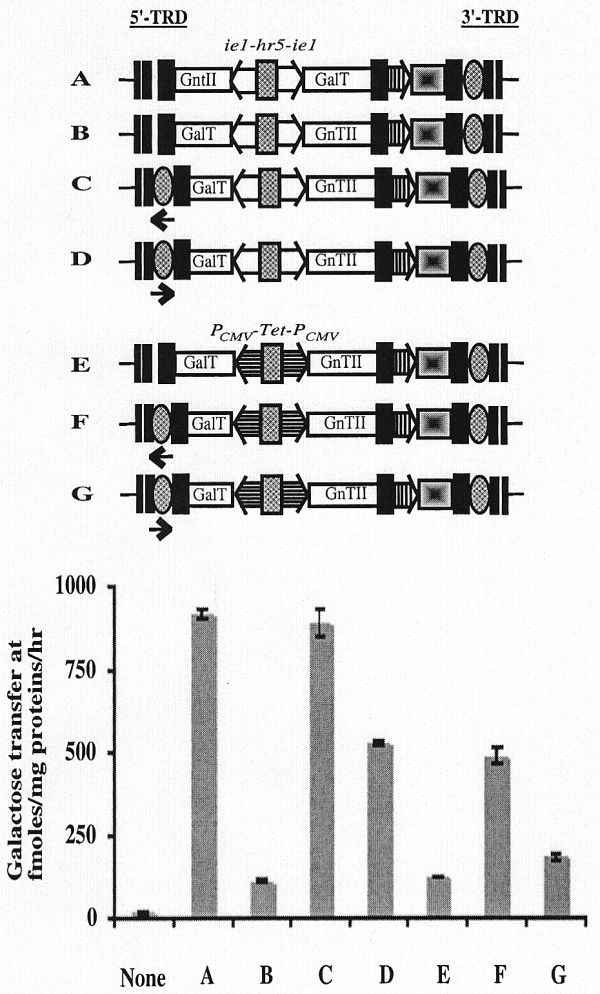
**The *cis*-activating function of the 3'-TRD internal domain sequence is essentially orientation-independent**. The genetic structures of the different *piggyBac *vectors assayed in this experiment (A-G) are shown above the plot. The dual constitutive (*ie1-hr5-ie1*) transcriptional control element is indicated by open boxes, the dual inducible (*P*_*CMVmin*_-TetO7-*P*_*CMVmin*_) transcriptional control element is indicated by horizontally striped boxes, the orientations of the various heterologous genes are shown, and the new 3'-TRD internal domain sequence introduced into the 5'-TRD region is marked with an cross-hatched oval and an arrow to show its orientation. The plot shows the relative levels of GalT activity induced by each of the indicated *piggyBac *vectors, together with the background levels determined using extracts of mock-transfected Sf9 cells (None).

Additional transient expression assays were performed to compare the influence of inserting a single copy of the 3'-TRD_ID _sequence into the constitutive and inducible *piggyBac *derivatives downstream and in the same (Fig. 10, vectors C and F) or opposite (Fig. 10, vectors D and G) orientation, relative to the leftward-facing GalT gene. Constitutive and inducible *piggyBac *vectors containing the GalT gene in the leftward orientation with no downstream copy of the 3'-TRD_ID _sequence (Fig. 10, vectors B and E) were used as negative controls and a constitutive *piggyBac *vector containing the GalT gene in the rightward orientation (Fig. 10, vector A) was used as a positive control. The results of these assays showed that addition of the 3'-TRD_ID _sequence downstream of the leftward facing GalT genes induced higher activity levels, irrespective of its orientation, relative to the negative controls lacking the downstream 3'-TRD_ID _sequence (Fig. 10). This effect was not completely orientation-independent, however, as the *piggyBac *vectors containing the 3'-TRD_ID _sequence in the same orientation as the leftward facing GalT gene (vectors C and F) induced higher levels of GalT activity than those containing the 3'-TRD_ID _sequence in the opposite orientation (vectors D and G).

### Functionality of dual piggyBac vectors in transgenic insects

**Figure 11:**
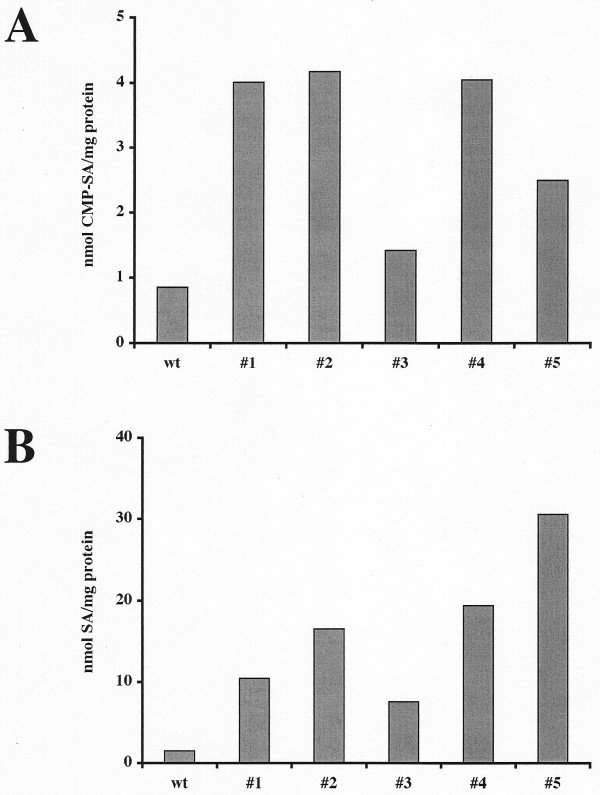
**A representative dual, constitutive *piggyBac *vector is functional as an insect transformation vector**. *D. melanogaster *was transformed with pXLBacII-CMP-SAS/SAS-EYFP.LTR-F cl 25, which encodes CMP-SAS and SAS under the control of the *ie1-hr5-ie1 *dual constitutivetranscriptional control element, and several transgenic lines were isolated, as described in Methods. After being cultured in the presence of *N*-acetylmannosamine, larvae from five transgenic lines or wild type were extracted and the extracts were used to measure total CMP-sialic acid (A) and sialic acid (B) contents, as described in Methods. Each bar represents the average results obtained using duplicate samples of extracts from groups of larvae obtained from each fly line.

**Figure 12:**
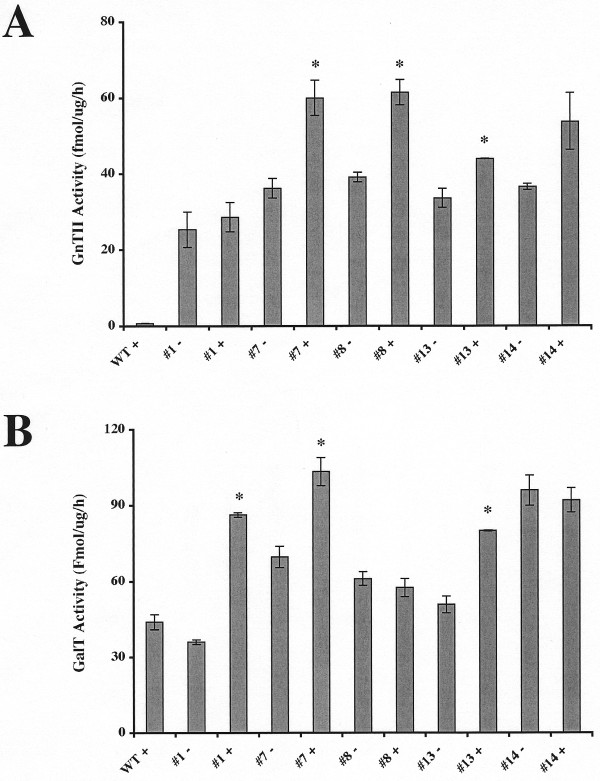
**A representative dual, inducible *piggyBac *vector is functional as an insect transformation vector**. *D. melanogaster *was transformed with pXLBacII-TetO1.GalT/GnTII-DsRed.A cl 3, which encodes GalT and GnTII under the control of the *P*_*CMVmin*_-*TetO7*-*P*_*CMVmin *_dual tetracycline-inducible transcriptional element, and several transgenic lines were isolated, as described in Methods. After being cultured in the presence or absence of doxycycline, larvae from five transgenic lines or wild type were extracted and the extracts were used to measure GnTII (A) and GalT (B) activities, as described in Methods. Each bar represents the average results obtained using duplicate samples of extracts from groups of larvae obtained from each fly line. The error bars show the standard deviations and the asterisks mark the lines that had statistically significant (p = 0.05) differences in enzyme activity levels measured in the absence and presence of doxycycline. The statistical analysis represents the results of one-tailed student's t-tests with the assumption of equal variances, which was checked by comparing the ratio of the variances with the appropriate F value on a table of F values for p = 0.05. The equal variance assumption failed for the GnTII (panel A) assays on lines #13 and #14 and for the GalT assay on line #13. Thus, those t-tests were repeated assuming unequal variance and these results are reported. The p values obtained for the GnTII assays (A) were #1 (0.26), #7 (0.01), #8 (0.01), #13 (0.05), and #14 (0.10). The p values obtained for the GalT assays (B) were #1 (0.00), #7 (0.01), #8 (0.19), #13 (0.03), and #14 (0.27).

Finally, we examined the insect transformation functions of two representative members of our large new set of dual *piggyBac *vectors, one designed for constitutive expression and the other designed for inducible expression of heterologous gene pairs. In the first experiment, *D. melanogaster *was transformed with one of our dual *piggyBac *vectors encoding SAS and CMP-SAS under the control of the dual constitutive transcriptional control element. Five transformed fly lines were then fed with *N*-acetylmannosamine, larval homogenates were prepared, and sialic acid and CMP-sialic acid contents were assayed, as described in Methods. The results showed that this representative *piggyBac *vector could, indeed, be used to transform an insect for constitutive expression of this heterologous gene pair, as the transformed fly lines all had high levels of both free sialic acid and CMP-sialic acid than the wild type control (Fig. 11). In the second experiment, *D. melanogaster *was transformed with one of our dual *piggyBac *vectors encoding GalT and GnTII under the control of the dual inducible transcriptional control element. Five transformed fly lines were then fed with or without doxycycline, larval homogenates were prepared, and GalT and GnTII activities were assayed, as described in Methods. The results showed that all of the fly lines had higher levels of GnTII activity and all but one had higher levels of GalT activity than the wild type controls (Fig. 12), which indicated that the inducible control element is not tightly regulated in transgenic insects. However, doxycycline induced GnTII and GalT activities in 4/5 and 3/5 lines examined, respectively. Thus, while undetermined factors, such as the nature of the integration site, can influence its function, these data show that the representative dual *piggyBac *vector designed for inducible expression of heterologous gene pairs was functional in transgenic insects.

## Conclusion

The initial purpose of this study was to produce and characterize new *piggyBac *vectors that could be used to transfer heterologous gene pairs into the genome of a target organism in either constitutively- or inducibly-expressible forms. Functional characterization of these new vectors revealed an unexpected position effect that was independent of the identity of the heterologous gene or the transcriptional control element. In the process of performing experiments designed to help us understand the underlying reason for this position effect, we discovered a previously unrecognized *cis-*activating element derived from the internal domain of the 3' terminal repeat in the *piggyBac *transposable element. This element appears to function as an enhancer element, as it stimulates heterologous gene expression in an essentially orientation-independent fashion, albeit to different levels. However, formal definition of this element as an enhancer would require additional characterization to determine if it can function autonomously in a position- and orientation-independent fashion.

The discovery of this element will be of general interest to investigators who are aware of *piggyBac *and its widespread and growing applications as a transformation vector. In addition, given their ability to provide constitutive or inducible expression of heterologous gene pairs, the large set of new dual *piggyBac *vectors described in this study will be of interest to investigators who need to introduce multiple genes into a single target organism.

## Methods

### PCR amplification

High fidelity *KOD *(Novagen, Madison, WI) DNA polymerase was used as described in the manufacturer's manual. Briefly, 50 μL PCR reactions consisted of 5 μL of 10X *KOD *DNA polymerase buffer, 5 μL of dNTP mix (10 mM each), 2 μL of 25 mM MgCl_2_, 0.5 μL of *KOD *DNA polymerase, 0.5 μL of each primer (50 μM), 10 μL of template, and 26.5 μL of H_2_O. The fragments were amplified after an initial denaturation step at 95°C for 2 min using 30 cycles of 15 sec at 98°C, 30 sec at appropriate primer annealing temperatures, and 1.5 min at 72°C. The desired fragments were purified by agarose gel fractionation before being cloned into either pCRBluntII-TOPO (Invitrogen, Carlsbad, CA) or pCR2.1-TOPO (Invitrogen), and sequence-verified clones were used to assemble all of the final constructs described in this study.

### Molecular cloning

General molecular cloning methods were performed as described in reference [19]. PCR products were cloned into pCRBluntII-TOPO (Invitrogen) or pCR2.1-TOPO as described by the manufacturer. All restriction endonucleases were purchased from New England Biolabs, Inc. (Beverly, MA).

### Construction of piggyBac vectors for constitutive expression of heterologous gene pairs

A transcriptional control element consisting of back-to-back baculovirus *ie1 *promoters separated by a baculovirus *hr5 *enhancer was constructed in a series of steps (Fig. 1, 2, 3, 4), which began with PCR amplification of DNA fragments termed hr5IE1R and IE1L using pAcP(+)IE1TV3 [20] as the template and Hr5IE1Rsense plus Hr5IE1Ranti or IE1Lsense plus IE1Lanti as the primers (Table 1 and Fig. 1A). Each of the resulting amplification products was cloned into pCRBluntII-TOPO (Invitrogen) and error-free clones identified by restriction mapping and DNA sequencing were designated pHr5IE1R-TOPO.1 and pIE1L-TOPO.1. The desired "*ie1-hr5-ie1*" dual constitutive transcriptional control element was subsequently assembled by excising the IEL fragment from pIE1L-TOPO.1 with *Xba*I and *Kpn*I and subcloning it into the *Spe*I and *Kpn*I sites of pHr5IE1R-TOPO.1, which yielded pDIE1-TOPO.1. The *Xba*I site in pDIE1-TOPO.1 was then ablated by *Xba*I digestion, Klenow repair, and re-ligation to produce pDIE1-TOPO.2.

**Table 1:** Oligonucleotides used in this study

**Name**	**T**_**m**_	**Sequence (5' to 3')**
Hr5IE1Rsense	55°C	CGCGTAAAACACAATCAAG
HR5IE1Ranti	73°C	TCGCGAGGTCACTTGGTTGTTCACG
IE1Lsense	70°C	TCTAGACGATGTCTTTGTGATGCGCG
IE1Lantsense	69°C	GGTACCGGTCACTTGGTTGTTCACG
BGHsense	89°C	GGTACCGTTTAAACGGATCCCTCGAGACTAGTGGGCCCGCCTCGACTG
BGHanti	83°C	AAGCTTATAACGGAGACCTCTAGAGGATCCTCCCCAGCATGCCTGCTATTG
3xP3sense	63°C	AGATCTTAATTCAATTAGAGACTAATTC
3xP3anti	65°C	GCTAGCGATTGTTTAGCTTGTTCAGC
DsRed1sense	74°C	GCTAGCATGGTGCGCTCCTCCAAGAA
DsRed1anti	75°C	GGGCCCCTACAGGAACAGGTGGTTGGC
ECFPsense	75°C	GCTAGCATGGTGAGCAAGGGCGAGGAG
ECFPanti	72°C	GGGCCCTTACTTGTACAGCTCGTCCATG
EYFPsense	75°C	GCTAGCATGGTGAGCAAGGGCGAGGAG
EYFPanti	72°C	GGGCCCTTACTTGTACAGCTCGTCCATG
HumanGlcNAcTIIsense	71°C	GTTTAAACACCATGAGGTTCCGCATCTACAAA
HumanGlcNAcTIIanti	55°C	ACTAGTTCACTGCAGTCTTCTATAAC
bovineβ4GalTsense	82°C	TCGCGAACCATGAAGTTTCGGGAGCCGCTC
bovineβ4GalTanti	86°C	GCGGCCGCCTAGCTCGGCGTCCCGATGTC
ratST6sense	66°C	GTTTAAACACCATGATTCATACCAACTTGAAG
ratST6anti	67°C	ACTAGTTCAACGAATGTTCCGGAAG
mouseST3sense	76°C	TCGCGAACCATGACCAGCAAATCTCACTG
mouseST3anti	76°C	GCGGCCGCTCAGAAGTATGTGAGGTTCTTG
mouseSASsense	77°C	GTTTAAACACCATGCCGCTGGAACTGGAGCTG
MouseSASanti	60°C	ACTAGTTTAAGCCTTGATTTTCTTGC
mouseCMP.SASsense	84°C	GCGGCCGCCTATTTTTGGCATGAGTTATTAAC
mouseCMP.SASanti	75°C	TCGCGAACCATGGACGCGCTGGAGAAGGG
PmeCMV	73°C	GTTTAAACAGGCTGGATCGGTCCCGG
NruCMV	80°C	GTCGCGAAGGCTGGATCGGTCCCGG
HpaTetO	60°C	GTTAACTTTACCACTCCCTATCAGTGA
HpaTetO2	66°C	GTTAACGGACTTTCCACACCTCGAG
HpaCMV	69°C	GTTAACCTCGGTACCCGGGTCGAG
BGHpolyASeq1	60°C	CGGTGGAGCTCCAGCTT
BGHpolyASeq2	61°C	AACGACCGCGTGAGTCAA
5'LTR_ID_	59°C	CTAGAGATCTTGTTTTATCGGTCTGTA
3'LTR_ID_	59°C	CTAGAGATCTCAGGAATTCGATAAAAG

In a parallel set of cloning reactions (Fig. 1B), a DNA fragment containing the bovine growth hormone polyadenylation signal [21] was PCR amplified using pCR3.1 (Invitrogen) as the template and BGHsense and BGHanti as the primers (Table 1). The product was cloned into pCRBluntII-TOPO and an error-free clone identified by restriction mapping and DNA sequencing was designated pBGHpolyA-TOPO.1. The BGHpolyA fragment was then excised from pBGHpolyA-TOPO.1 with *Apa*I and *Bsa*I and subcloned into the same sites of pDIE1-TOPO.2 to produce pDIE1-TOPO.3.

A third parallel set of cloning reactions (Fig. 2A) was performed to place three different fluorescent marker genes under the transcriptional control of the eye-specific promoter, 3xP3 [22], for subsequent insertion into pDIE1-TOPO.3. First, the 3xP3 promoter fragment was PCR amplified using pBSII-LTR1.1k-ECFP [18] as the template and 3xP3sense plus 3xP3anti as the primers (Table 1). The product was cloned into pCRBluntII-TOPO, an error-free clone designated p3xP3-TOPO.1 was identified by restriction mapping and DNA sequencing, and then the *Apa*I and *Bsa*I fragment of pBGHpolyA-TOPO.1, which contains the BGH polyA signal, was inserted at the corresponding sites of this plasmid to produce p3xP3-TOPO.2. Subsequently, each fluorescent marker gene of interest, including DsRed1, EGFP, and EYFP, was individually PCR-amplified using p3xP3-DsRed1-ORF (Li and Fraser, unpublished), pBSII-LTR1.1k-ECFP [18], and pBSII-LTR1.1k-EYFP [18] as the templates and DsRed1sense plus DsRed1anti, EGFPsense plus EGFPanti, and EYFPsense plus EYFPanti (Table 1) as the primers, respectively. Each individual PCR product was cloned into pCRBluntII-TOPO to produce pDsRed1-TOPO.1, pECFP-TOPO.1, and pEYFP-TOPO.1, respectively, and error-free clones were identified by restriction mapping and DNA sequencing. Finally, each individual marker was excised with *Nhe*I and *Apa*I and subcloned into the corresponding sites of p3xP3-TOPO.2 to produce p3xP3DsRed1-TOPO.2, p3xP3ECFP-TOPO.2, and p3xP3EYFP-TOPO.2.

The eye color markers were subsequently incorporated into the intermediate plasmids containing the dual constitutive *ie1-hr5-ie1 *transcriptional control element (Fig. 2B). The red, cyan, and yellow fluorescent protein markers were excised by digesting 3xP3DsRed1, 3xP3ECFP, and 3xP3EYFY with *Bgl*II and *Bsa*I and each was individually subcloned into the *Bam*HI and *Bsa*I sites of pDIE1-TOPO.3 to produce pDIE1DsRed1-TOPO.3, pDIE1ECFP-TOPO.3, and pDIE1EYFP-TOPO.3, respectively. Subsequently, the *Kpn*I-*Hind*III fragment from pBGHpolyA-TOPO.1 was subcloned into the corresponding sites downstream of the leftward *ie1 *promoter in each of these plasmids. This yielded key intermediate plasmids designated pDIE1DsRed1-TOPO.4, pDIE1ECFP-TOPO.4, and pDIE1EYFP-TOPO.4, each of which included the *ie1-hr5-ie1 *regulatory element for constitutive expression of heterologous gene pairs, unique restriction sites downstream of both promoters for insertion of the heterologous genes of interest, and a fluorescent eye color marker for the identification of transgenic offspring.

For the purposes of a project that will be described elsewhere, we subsequently inserted six heterologous mammalian genes encoding enzymes involved in protein *N*-glycosylation into the three key intermediate plasmids described above (Fig. 3). A human *N*-acetylglucosaminyltransferase II (GnTII; [23]) coding sequence was PCR amplified using pHG30 [23] as the template and humanGlcNAcTIIsense plus humanGlcNAcTIIanti (Table1) as the primers. Similarly, the sequence encoding bovine β1,4-galactosyltransferase (GalT; [24]) was amplified using pBSKS-β4GalT as the template and bovineβ 4GalTsense plus bovineβ4GalTanti (Table 1) as the primers, the sequence encoding a rat α2,6-sialyltransferase (ST6GalI; [25]) was amplified using pIE1HR3ST6Δcys [26] as the template and ratST6sense plus ratST6anti (Table 1) as the primers, the sequence encoding a mouse α2,3-sialyltransferase (ST3GalIII; [27,28]) was amplified using pST3GalIII [28] as the template and mouseST3sense plus mouseST3anti (Table 1) as the primers, the sequence encoding mouse sialic acid synthase (SAS; [29]) was amplified using p64KDIE1TV1/SAS/CMP-SAS [30] as the template and mouseSASsense plus mouseSASanti as the primers, and the sequence encoding mouse CMP-sialic acid synthetase (CMP-SAS;[31]) was amplified using p64KDIE1TV1/SAS/CMP-SAS [30] as the template and mouseCMP.SASsense plus mouseCMP.SASanti (Table 1) as the primers. Except for ST3GalIII, each amplification product was cloned into pCRBluntII-TOPO (Invitrogen) and error-free clones were identified by DNA sequencing. The ST3GalIII product had two single nucleotide substitutions at amino acid positions 80 (phenylalanine to serine) and 294 (glycine to cysteine), which were corrected by site-directed mutagenesis before subcloning, and the corrected amplification product was designated ST3.3m. Subsequently, *Pme*I-*Spe*I fragments encoding GnTII, ST6GalI, or SAS (designated as "Glyco-A" genes in Fig. 3) were inserted at the corresponding sites downstream of the leftward *ie1 *promoters and *Nru*I-*Not*I fragments encoding GalT, ST3.3m, or CMP.SAS ("Glyco-B" genes in Fig. 3) were inserted at the corresponding sites located downstream of the rightward *ie1 *promoters in the relevant intermediate plasmids. This yielded three derivatives termed pDIE1-GnTII/GalT-DsRed1-TOPO.4, pDIE1-ST6.1/ST3.3-ECFP-TOPO.4, and pDIE1-SAS/CMP.SAS-EYFP-TOPO.4. Finally, the expression cassettes were excised from these derivatives with *Xba*I and each was individually subcloned into the corresponding site of the *piggyBac *vector, pXLBacII [18]. This final step yielded six *piggyBac *vectors, designated pXLBacII-GnTII/GalT-DsRed1, pXLBacII-ST6.1/ST3.3-ECFP, and pXLBacII-SAS/CMP.SAS-EYFP, which contained the three different constitutive expression cassettes in each of the two different orientations. The three vectors containing the expression cassettes with the "Glyco-A" genes facing rightward (pXLBacII-GalT/GnTII-DsRed1.A cl 57, pXLBacII-ST3.3/ST6.1-ECFP.A cl 29, and pXLBacII-CMP.SAS/SAS-EYFP.A cl 42) were designated as vector "Set 1" and those containing these same genes facing leftward (pXLBacII-GnTII/GalT-DsRed1.B cl 1, pXLBacII-ST6.1/ST3.3-ECFP.B cl 21, and pXLBacII-SAS/CMP.SAS-EYFP.B cl 1) were designated as vector "Set 2" (Fig. 3).

### Construction of piggyBac vectors for inducible expression of heterologous gene pairs

A transcriptional control element consisting of back-to-back minimal cytomegalovirus immediate early promoters (*P*_*CMVmin*_) separated by a tetracycline-inducible operator (*TetO7*; [32]) was constructed in several steps (Fig. 4). First, copies of the *P*_*CMVmin *_element and the fused *TetO7-P*_*CMVmin *_element were individually PCR amplified using pTRE2hyg-luc (BD Biosciences, Palo Alto, CA) as the template and HpaCMV plus NruCMV or HpaTetO plus PmeCMV as the primers (Table 1). Each of the resulting DNA fragments was cloned into pCR2.1-TOPO (Invitrogen) and error-free clones identified by restriction mapping and DNA sequencing were designated pCR2.1-HpaCMVNru and pCR2.1-PmeTetO, respectively. Subsequently, the *P*_*CMVmin *_fragment was excised from pCR2.1-HpaCMVNru with *Hpa*I and *Bam*HI and subcloned into the corresponding sites of pCR2.1-PmeTetO to produce pCR2.1-NruTetOPme, which contained the fully assembled control element consisting of back-to-back copies of the CMV promoter separated by the tetracycline-inducible operator. This "*P*_*CMVmin*_-*TetO7*-*P*_*CMVmin*_" regulatory element was then excised from pCR2.1-NruTetOPme with *Nru*I and *Pme*I and used to replace the *"ie1-hr5-ie1" *control element in a subset of the dual constitutive *piggyBac *vectors from Sets 1 and 2, which were described above. This yielded two new sets of *piggyBac *vectors (Sets 3 and 4), which were designated pXLBacII-TetO1.GalT/GnTII-DsRed.A cl 3, pXLBacII-TetO1.CMP-SAS/SAS-EYFP.A cl 35, pXLBacII-TetO1.GnTII/GalT-DsRed.B cl 18, and pXLBacII-TetO1.ST6.1/ST3.3-ECFP.B cl 30. These vectors encoded the indicated heterologous gene pairs under the control of the tetracycline inducible transcriptional control element, as well as the various fluorescent eye color markers described above.

### Construction of BGHpolyA-modified piggyBac vectors

Two of the *piggyBac *vectors in Set 1 described above were further modified by the insertion of an additional BGHpolyA signal downstream of the 5'-TRD. A DNA fragment containing the BGH polyA signal was excised from pBGHpolyA-TOPO.1 (Fig. 1B) with *Apa*I and *Bam*HI, the ends were repaired with Klenow, and the resulting blunt ended DNA fragment was inserted into the Klenow-repaired *Bgl*II site in the internal domain in the 5'-TRDs of pXLBacII-GalT/GnTII-DsRed1.A cl57 and pXLBacII-TetO1.GalT/GnTII-DsRed1.A cl 3 (Fig. 4 and Fig. 6, top). The two desired BGHpolyA-modified vectors, which had the additional BGHpolyA signal oriented in the same direction as the remnant *piggyBac *promoter element in the 5'-TRD (vectors E and F in Fig. 6, top), were identified by colony PCR with the primer pair BGHpolyASeq1 plus BGHsense (Table 1) and DNA sequencing. These two new vectors were designated pXLBacII-GalT/GnTII-DsRed1.BGH.A cl 2 and pXLBacII-TetO1.GalT/GnTII-DsRed1.BGH.A cl 20.

### Construction of piggyBac vectors lacking the transposase promoter

Two additional *piggyBac *vectors lacking the *piggyBac *transcription start region (TSR) in the 5'-TRD [4,18] were constructed by digesting pXLBacII with *Hind*III and *Sph*I to delete nucleotides 1146-1429, repairing the ends with Klenow, and religating to produce a derivative designated pXLBacIIΔTSR. Subsequently, the constitutive GnTII/GalT expression cassette was inserted at a unique *Xba*I site and clones containing the insert in either orientation were identified by restriction mapping and designated pXLBacIIΔTSR-GalT/GnTII/-DsRed1.A cl 16 and pXLBacIIΔTSR-GnTII/GalT-DsRed1.B cl 13.

### Construction of 3'-TRDID-modified piggyBac vectors

The final *piggyBac *vectors constructed for this study were designed to have an additional copy of a highly AT-rich (83% A+T), putative transcriptional activator derived from the *piggyBac *3' internal domain [4,18]. A DNA fragment containing nucleotides 789-986 of pXLBacII, which contained this putative transcriptional activator, was produced by PCR with pXLBacII as the template and 5'LTRactivator plus 3'LTRactivator as the primers (Table 1). The resulting PCR fragment was cloned into the Klenow-repaired *Bgl*II site downstream of the leftward-facing GalT gene in pXLBacII-GalT/GnTII-DsRed1.A cl 57. Derivatives containing the insert in either the forward or reverse orientation, with respect to the GalT gene, were identified by restriction mapping and DNA sequencing and designated pXLBacII-GalT/GnTII-DsRed1.LTR.F cl 16 and pXLBacII-GalT/GnTII-DsRed1.LTR.R cl 25, respectively. Analogous derivatives of the inducible *piggyBac *vectors were produced by inserting the blunt-ended DNA fragment containing the putative transcriptional activator sequence into the Klenow-repaired *Bgl*II sites of pXLBacII-TetO1.GalT/GnTII-DsRed1.A cl 3 and identifying clones containing the insert in either the forward or reverse orientation with respect to the GalT gene, which were designated pXLBacII-TetO1.GalT/GnTII-DsRed1.LTR.F cl 23 and pXLBacII-TetO1.GalT/GnTII-DsRed1.LTR.R cl 24, respectively. We also cloned this same putative transcriptional activator fragment in the forward orientation, with respect to the leftward-facing CMP-SAS and ST6GalI genes, using the Klenow-repaired *Bgl*II sites of pXLBacII-CMP-SAS/SAS-EYFP.A cl 42 and pXLBacII-TetO1.CMP-SAS/SAS-EYFP.A cl 35 and the Klenow-repaired *Sal*I sites of pXLBacII-ST6.1/ST3.3-ECFP.B cl 1 and pXLBacII-TetO1.ST6.1/ST3.3-ECFP.A cl30, respectively. This yielded pXLBacII-ST6.1/ST3.3-ECFP.LTR.F cl 10, pXLBacII-TetO1.ST6.1/ST3.3-ECFP.LTR.F cl 11, pXLBacII-CMP-SAS/SAS-EYFP.LTR-F cl 25, and pXLBacII-TetO1.CMP-SAS/SAS-EYFP.LTR-F cl 12, which completed the set of 3'-TRD-modified *piggyBac *vectors required for our studies. The sequence of the newly inserted putative activator fragment was directly confirmed by DNA sequencing of each of the 3'-TRD_ID_-modified *piggyBac *vectors.

### Glycosyltransferase assays

Cultures containing 2 × 10^6 ^Sf9 cells were transfected with 10 μg of the relevant plasmid DNA(s) using a modified calcium phosphate precipitation method [33]. At 24 h post-transfection, the cells were washed once with ice-cold Tris-buffered saline (TBS; 50 mM Tris.Cl, pH7.5, and 150 mM NaCl) and once with the buffer to be used for the relevant glycosyltransferase activity assay. The cells were then extracted with the same glycosyltransferase buffer supplemented with 1% (v/v) Triton X-100 (Sigma-Aldrich, St. Louis, MO) and the extracts were frozen prior to being used for the glycosyltransferase assays. The tetracycline-inducible *piggyBac *vectors were assayed using a slightly different method in which Sf9 cells were co-transfected with the vector of interest in the presence of a helper plasmid, pBSK.Hr5IE1.rtTAM2.SV40-1.1k.ITR-3xP3-EGFP, which encodes the mutant repressor needed to induce transcription in the presence of tetracycline. At 12 h post-transfection, these cell cultures were treated with fresh growth medium containing 1.0 μg/mL doxycycline (BD Biosciences) and they were extracted 24 h later.

The GnTII, GalT, ST6, and ST3 enzyme activity assays were performed as described previously [16,26,28,34]. The cell extraction and enzyme assay buffers used for these experiments were GnTII buffer [100 mM MES, pH 6.1, 100 mM NaCl and 1% (v/v) Triton X-100], GalT buffer (10 mM HEPES, pH 7.4, 140 mM NaCl, 20 mM MnCl_2_, and 0.5% Nonidet P-40), ST6 buffer (50 mM Na_2_HPO_4_, pH 7.5, 100 mM NaCl, 10 mM MgCl_2_, and 1.5% Triton CF-54), and ST3 buffer (100 mM sodium cacodylate, pH 6.4, 10 mM MgCl_2_, 2 mM CaCl_2_, and 1.5% Triton CF-54). Cells were washed with these buffers minus the detergents and extracted with these buffers plus the detergents, as described above, and the extracts were frozen at -85°C. Prior to performing the assays, the cell extracts were thawed, clarified at 1,000 × *g *for 5 min at 4°C in a microcentrifuge, and total protein concentrations were determined using a commercial bicinchoninic acid assay (Pierce, Rockford, IL) with BSA as the standard. Duplicate samples of each extract, containing 100 μg of total protein, were then incubated at 37°C for 1 h with donor and acceptor substrates in the appropriate buffers and supplements. The final GnTII assay reaction contained 67 mM MES (pH 6.1), 67 mM NaCl, 15 mM MnCl_2_, 6.7 mM AMP, 133 mM *N*-acetylglucosamine, 0.0833 mM Manα1,6(GlcNAcβ1,2Manα1,3)-Manβ-octyl (Toronto Research Chemicals, Ontario, Canada), and 0.9 μCi of uridine diphosphate [6-^3^H]-*N*-acetylglucosamine (60 Ci/mmol; American Radiolabeled Chemicals, Inc., St. Louis, MO). The final GalT assay reaction contained 0.3 μCi of uridine diphosphate [6-^3^H]-galactose (9.1 Ci/mmol; American Radiolabeled Chemicals) and 830 ug/mL of ovalbumin (Sigma-Aldrich) in the GalT buffer described above. The final ST6GalI and ST3GalIII assay reactions contained 0.3 μCi of cytidine 5'-monophosphate [6-^14^C] sialic acid (20 Ci/mmol; American Radiolabeled Chemicals) and 310 ug/mL of asialofetuin (Sigma-Aldrich). After the 1 h incubation period, each reaction was quenched by dilution with ice cold water and radiolabeled GnTII products were collected by reverse phase chromatography with SepPak C18 cartridges (Millipore, Bedford, MA), while radiolabeled GalT, ST6GalI, and ST3GalIII products were collected by TCA precipitation onto Whatman GF/D glass microfibre filters (Whatman Inc., Florham Park, N.J.). Following elution, the amounts of radioactivity transferred to each donor substrate in duplicate reactions were measured with a Model LS-6500 liquid scintillation spectrometer (Beckman-Coulter Instruments, Palo Alto, CA), the results were averaged, and the average values were converted to the average fmol of donor substrate transferred/μg total protein/h using the specific radioactivities of the donor substrates.

### Sialic acid and CMP-sialic acid assays

SAS and CMP-SAS activities were determined by measuring sialic acid and CMP-sialic acid levels in transfected cell lysates, as described previously [35,36]. Briefly, Sf9 cells were pre-incubated for 12 h with growth medium containing 10 mM *N-*acetylmannosamine with or without 1.0 μg/mL doxycycline. The cells were then transfected with the relevant plasmid DNAs, incubated for another 24 h, rinsed twice with ice-cold TBS buffer, and lysed in cold TBAS buffer (0.2 M Tris. pH 9.0, 0.2 mM DTT, 20 mM MgCl_2_, 1% Triton X-100). The lysates were clarified, total protein concentrations were determined as described above, and duplicate assays were performed with samples containing 1.0 mg of total protein. For the CMP-sialic acid determinations, the cell lysate was pre-treated with 50 μL of 1.6 M NaBH_4 _to reduce the free sialic acid and then with 55 μL of concentrated H_3_PO_4 _to destroy the excess NaBH_4 _and hydrolyze the CMP-sialic acid. The released sialic acid was oxidized by adding 50 μL of 0.2 M NaIO_4 _and the excess NaIO_4 _was subsequently destroyed by adding 0.4 mL of 4% NaAsO_4 _in 0.5 M HCl. After vigorous vortexing to eliminate the brown color, 2 mL of 0.1 M 2-thiobarbituric acid (adjusted to pH 9.0 with NaOH) were added and the reaction mixtures were incubated in a 100°C waterbath for 7.5 min to generate the pink chromophore, which was extracted overnight at room temperature with 4 mL of *n*-butanol containing 0.6 N HCl. Finally, the organic phase was collected, absorbance was measured at 532 nm, 549 nm, and 562 nm, and nmol CMP-sialic acid was calculated using a standard conversion factor (21 × OD_549 nm _- 7.58 × OD_532 nm_) × 4.0 [35]. The method used to measure total sialic acid content was the same as described above except the cell lysates were not pre-treated with NaBH_4_. The results were expressed as sialic acid or CMP-sialic acid content/μg total protein.

### Isolation and analysis of transgenic insects

The representative *piggyBac *vectors used to produce transgenic insects were pXLBacII-CMP-SAS/SAS-EYFP.LTR-F cl 25, which encodes CMP-SAS and SAS under the control of the *ie1-hr5-ie1 *dual constitutive transcriptional control element, and pXLBacII-TetO1.GalT/GnTII-DsRed.A cl 3, which encodes GalT and GnTII under the control of the *P*_*CMVmin*_-*TetO7*-*P*_*CMVmin *_dual tetracycline-inducible transcriptional element. Drosophila strains were reared under standard laboratory conditions [37]. *D. melanogaster w*^1118 ^white eye pre-blastoderm embryos were microinjected as described previously, except there was no dechorionation step [38]. pXLBacII-CMP-SAS/SAS-EYFP.LTR-F cl 25 was injected at a concentration of 0.5 ug/uL together with 0.3 ug/uL of phspBac, which encodes the *piggyBac *transposase, while pXLBacII-TetO1.GalT/GnTII-DsRed.A cl 3 was injected at a concentration of 0.6 ug/uL together with 0.4 ug/uL of phspBac. To produce a fly line encoding the transcription factor needed for tetracycline induction, pBS.rtTAM2-EGFP was co-injected together with phspBac at equal concentrations of 0.4 ug/uL. One day later, all microinjected embryos were subjected to a one hour heat shock at 37°C to induce expression of the *piggyBac *transposase, and they were subsequently reared at 28°C. Emerging adults were individually mated with w^1118 ^flies, and their progeny were screened for fluorescent eye color as adults using an Olympus SZX12 fluorescent microscope equipped with YFP and RED filter sets. Positive adults were individually crossed with the w^1118 ^flies and subsequent generations were produced to establish each separate, homozygous transgenic fly line. Constitutive expression of SAS and CMP-SAS was examined by mating individual adults from the relevant homozygous fly lines, allowing the females to lay eggs on diet with or without 10 mM *N*-acetylmannosamine, and rearing the hatched larvae through fourth instar on the same diets. These larvae were then homogenized in ice-cold TBA buffer and the homogenates were clarified and used to measure total protein concentrations and sialic acid and CMP-sialic acid contents, as described above. To produce transgenic fly lines capable of inducibly expressing GnTII and GalT, homozygous adults encoding the rtTAM2 transcription factor were mated with homozygous adults encoding GnTII and GalT under the control of the dual tet-inducible CMV promoter. Progeny were screened for expression of both EGFP and DsRed and then single males and single virgin females from each cross were mated. The females were placed into vials containing diet with or without 50 ug/mL of doxycycline, allowed to lay eggs, and the hatched larvae were maintained on the same diet for ten days, homogenized in GnTII or GalT assay buffer, and the homogenates were clarified and used to measure total protein concentrations and GnTII and GalT activity levels, as described above.

## Competing interests

DLJ and MJF received funding for this project from the NIST-ATP program through Chesapeake-PERL, which could conceivably gain financially from publication of this manuscript. RLH and XS were directly employed by Chesapeake-PERL while working on this project. Chesapeake-PERL is not financing publication of this manuscript in any other way. DLJ, MJF, RLH, JRH, and XS are all inventors on a provisional patent application disclosing the properties of some of the *piggyBac *vectors described herein.

## Authors' contributions

DLJ and MJF conceived, designed, and coordinated the original project and provided scientific and administrative support. JRH developed the detailed construction plan for the *piggyBac *vectors and produced some of the early constructs. RLH constructed and characterized the original dual *piggyBac *vectors and performed the experiments that revealed the position effect. XS constructed and characterized the remainder of the *piggyBac *vectors, performed the experiments that revealed why there was a position effect, assayed the transgenic fly extracts, and drafted the manuscript. AM isolated and characterized the transgenic frutflies. DLJ extensively revised the manuscript and MJF and RLH suggested substantial revisions, which were incorporated by DLJ. All authors read and approved the final manuscript.

## References

[B1] Fraser MJ, Smith GE, Summers MD (1983). Acquisition of host cell DNA sequences by baculoviruses:  Relationship between host DNA insertions and FP mutants of Autographa californica and Galleria mellonella nuclear polyhedrosis viruses. J Virol.

[B2] Fraser MJ, Brusca JS, Smith GE, Summers MD (1985). Transposon-mediated mutagenesis of a baculovirus. Virology.

[B3] Fraser MJ, Cary L, Boonvisudhi K, Wang HG (1995). Assay for movement of Lepidopteran transposon IFP2 in insect cells using a baculovirus genome as a target DNA. Virology.

[B4] Cary LC, Goebel M, Corsaro BG, Wang HG, Rosen E, Fraser MJ (1989). Transposon mutagenesis of baculoviruses: analysis of Trichoplusia ni transposon IFP2 insertions within the FP-locus of nuclear polyhedrosis viruses. Virology.

[B5] Fraser MJ, Ciszczon T, Elick T, Bauser C (1996). Precise excision of TTAA-specific lepidopteran transposons piggyBac (IFP2) and tagalong (TFP3) from the baculovirus genome in cell lines from two species of Lepidoptera. Insect Mol Biol.

[B6] Elick TA, Bauser CA, Fraser MJ (1996). Excision of the piggyBac transposable element in vitro is a precise event that is enhanced by the expression of its encoded transposase. Genetica.

[B7] Fraser MJ, James AA, Handler AH (2000). The TTAA-specific family of transposable elements. Insect Transgenesis: Methods and Applications.

[B8] Sarkar A, Sim C, Hong YS, Hogan JR, Fraser MJ, Robertson HM, Collins FH (2003). Molecular evolutionary analysis of the widespread piggyBac transposon family and related "domesticated" sequences. Mol Genet Genomics.

[B9] Handler AM, McCombs SD, Fraser MJ, Saul SH (1998). The lepidopteran transposon vector, piggyBac, mediates germ-line transformation in the Mediterranean fruit fly. Proc Natl Acad Sci U S A.

[B10] Handler AM (2002). Use of the piggyBac transposon for germ-line transformation of insects. Insect Biochem Mol Biol.

[B11] Balu B, Shoue DA, Fraser MJ, Adams JH (2005). High-efficiency transformation of Plasmodium falciparum by the lepidopteran transposable element piggyBac. Proc Natl Acad Sci U S A.

[B12] Ding S, Wu X, Li G, Han M, Zhuang Y, Xu T (2005). Efficient transposition of the piggyBac (PB) transposon in mammalian cells and mice. Cell.

[B13] Kost TA, Condreay JP, Jarvis DL (2005). Baculovirus as versatile vectors for protein expression in insect and mammalian cells. Nat Biotechnol.

[B14] Jarvis DL (2003). Developing baculovirus-insect cell expression systems for humanized recombinant glycoprotein production. Virology.

[B15] Aumiller JJ, Hollister JR, Jarvis DL (2003). A transgenic insect cell line engineered to produce CMP-sialic acid and sialylated glycoproteins. Glycobiology.

[B16] Breitbach K, Jarvis DL (2001). Improved glycosylation of a foreign protein by Tn-5B1-4 cells engineered to express mammalian glycosyltransferases. Biotechnol Bioengr.

[B17] Stebbins MJ, Urlinger S, Byrne G, Bello B, Hillen W, Yin JC (2001). Tetracycline-inducible systems for Drosophila. Proc Natl Acad Sci U S A.

[B18] Li X, Harrell RA, Handler AM, Beam T, Hennessy K, Fraser MJ (2005). piggyBac internal sequences are necessary for efficient transformation of target genomes. Insect Mol Biol.

[B19] Sambrook J, Fritsch EF, Maniatis T (1989). Molecular Cloning: A Laboratory Manual.

[B20] Jarvis DL, Weinkauf C, Guarino LA (1996). Immediate-early baculovirus vectors for foreign gene expression in transformed or infected insect cells. Protein Expr Purif.

[B21] Goodwin EC, Rottman FM (1992). The 3'-flanking sequence of the bovine growth hormone gene contains novel elements required for efficient and accurate polyadenylation. J Biol Chem.

[B22] Horn C, Jaunich B, Wimmer EA (2000). Highly sensitive, fluorescent transformation marker for Drosophila transgenesis. Dev Genes Evol.

[B23] Tan J, D'Agostaro AF, Bendiak B, Reck F, Sarkar M, Squire JA, Leong P, Schachter H (1995). The human UDP-N-acetylglucosamine: alpha-6-D-mannoside-beta-1,2-N-acetylglucosaminyltransferase II gene (MGAT2). Cloning of genomic DNA, localization to chromosome 14q21, expression in insect cells and purification of the recombinant protein. Eur J Biochem.

[B24] Shaper NL, Shaper JH, Meuth JL, Fox JL, Chang H, Kirsch IR, Hollis GF (1986). Bovine galactosyltransferase: identification of a clone by direct immunological screening of a cDNA expression library. Proc Natl Acad Sci USA.

[B25] Weinstein J, Lee EU, McEntee K, Lai PH, Paulson JC (1987). Primary structure of ß-galactoside alpha 2,6-sialyltransferase.  Conversion of membrane-bound enzyme to soluble forms by cleavage of the NH2-terminal signal anchor.. J Biol Chem.

[B26] Hollister J, Jarvis DL (2001). Engineering lepidopteran insect cells for sialoglycoprotein production by genetic transformation with mammalian ß1,4-galactosyltransferase and a2,6-sialyltransferase genes.. Glycobiology.

[B27] Weikert S, Papac D, Briggs J, Cowfer D, Tom S, Gawlitzek M, Lofgren J, Mehta S, Chisholm V, Modi N, Eppler S, Carroll K, Chamow S, Peers D, Berman P, Krummen L (1999). Engineering Chinese hamster ovary cells to maximize sialic acid content of recombinant glycoproteins. Nat Biotechnol.

[B28] Kono M, Ohyama Y, Lee YC, Hamamoto T, Kojima N, Tsuji S (1997). Mouse beta-galactoside alpha 2,3-sialyltransferases: comparison of in vitro substrate specificities and tissue specific expression. Glycobiology.

[B29] Nakata D, Close BE, Colley KJ, Matsuda T, Kitajima K (2000). Molecular cloning and expression of the mouse N-acetylneuraminic acid 9-phosphate synthase which does not have deaminoneuraminic acid (KDN) 9-phosphate synthase activity. Biochem Biophys Res Comm.

[B30] Aumiller JJ, Hollister JR, Jarvis DL (2003). A transgenic lepidopteran insect cell line engineered to produce CMP-sialic acid and sialoglycoproteins.. Glycobiology.

[B31] Munster AK, Eckhardt M, Potvin B, Muhlenhoff M, Stanley P, Gerardy-Schahn R (1998). Mammalian cytidine 5'-monophosphate N-acetylneuraminic acid synthetase: a nuclear protein with evolutionarily conserved structural motifs. Proc Natl Acad Sci U S A.

[B32] Gossen M, Freundlieb S, Bender G, Muller G, Hillen W, Bujard H (1995). Transcriptional activation by tetracyclines in mammalian cells. Science.

[B33] Summers MD, Smith GE (1987). A manual of methods for baculovirus vectors and insect cell culture procedures.. Tx Ag Expt Stn Bull No 1555.

[B34] Hollister J, Grabenhorst E, Nimtz M, Conradt H, Jarvis DL (2002). Engineering the protein N-glycosylation pathway in insect cells for production of biantennary, complex N-glycans. Biochemistry.

[B35] Warren L (1959). The thiobarbituric acid assay of sialic acids. J Biol Chem.

[B36] Vann WF, Silver RP, Abeijon C, Chang K, Aaronson W, Sutton A, Finn CW, Lindner W, Kotsatos M (1987). Purification, properties, and genetic location of Escherichia coli cytidine 5'-monophosphate N-acetylneuraminic acid synthetase. J Biol Chem.

[B37] Roberts DB (1998). Drosophila:  A Practical Approach.

[B38] Rubin GM, Spradling AC (1982). Genetic transformation of Drosophila with transposable element vectors. Science.

